# Wheat *TaNPSN SNARE* homologues are involved in vesicle-mediated resistance to stripe rust (*Puccinia striiformis* f. sp. *tritici*)

**DOI:** 10.1093/jxb/eru241

**Published:** 2014-06-24

**Authors:** Xiaodong Wang, Xiaojie Wang, Lin Deng, Haitao Chang, Jorge Dubcovsky, Hao Feng, Qingmei Han, Lili Huang, Zhensheng Kang

**Affiliations:** ^1^State Key Laboratory of Crop Stress Biology for Arid Areas and College of Plant Protection, Northwest Agriculture and Forestry University, Yangling, Shaanxi 712100, P. R. China; ^2^Department of Plant Science, University of California Davis, Davis, CA 95616, USA; ^3^Department of Biochemistry, Geisel School of Medicine at Dartmouth, Hanover, NH 03755, USA; ^4^Howard Hughes Medical Institute (HHMI), Chevy Chase, MD 20815, USA

**Keywords:** Bimolecular fluorescence complementation, immuno-localization, *Puccinia striiformis* f. sp. *tritici*, qRT-PCR, SNARE, vesicle-mediated resistance, virus-induced gene silencing, wheat, yeast two-hybrid.

## Abstract

Subcellular localisation of SNAREs (soluble N-ethylmaleimide-sensitive factor attachment protein receptors) and their ability to form SNARE complexes are critical for determining the specificity of vesicle fusion. *NPSN11*, a *Novel Plant SNARE* (*NPSN*) gene, has been reported to be involved in the delivery of cell wall precursors to the newly formed cell plate during cytokinesis. However, functions of *NPSN* genes in plant–pathogen interactions are largely unknown. In this study, we cloned and characterized three *NPSN* genes (*TaNPSN11*, *TaNPSN12*, and *TaNPSN13*) and three plant defence-related *SNARE* homologues (*TaSYP132*, *TaSNAP34*, and *TaMEMB12*). TaSYP132 showed a highly specific interaction with TaNPSN11 in both yeast two-hybrid and bimolecular fluorescence complementation (BiFC) assays. We hypothesize that this interaction may indicate a partnership in vesicle trafficking. Expressions of the three *TaNPSN*s and *TaSYP132* were differentially induced in wheat leaves when challenged by *Puccinia striiformis* f. sp. *tritici* (*Pst*). In virus-induced gene silencing (VIGS) assays, resistance of wheat (*Triticum aestivum*) cultivar Xingzi9104 to the *Pst* avirulent race CYR23 was reduced by knocking down *TaNPSN11*, *TaNPSN13* and *TaSYP132*, but not *TaNPSN12*, implying diversified functions of these wheat *SNARE* homologues in prevention of *Pst* infection and hyphal elongation. Immuno-localization results showed that TaNPSN11 or its structural homologues were mainly distributed in vesicle structures near cell membrane toward *Pst* hypha. Taken together, our data suggests a role of TaNPSN11 in vesicle-mediated resistance to stripe rust.

## Introduction

SNAREs (soluble N-ethylmaleimide-sensitive factor attachment protein receptors) are key components in vesicle trafficking in eukaryotic cells (Heese *et al.*, 2001; Wick *et al.*, 2003). Four different SNAREs form a SNARE complex to determine the specificity of intracellular fusion (Fukuda *et al.*, 2000). In detail, one helix of the SNARE bundle is formed by one vesicle membrane-anchored SNARE (v-SNARE) and three target membrane-anchored SNAREs (t-SNAREs) through their R-, Qa-, Qb- and Qc-SNARE domains (Antonin *et al.*, 2000; Fukuda *et al.*, 2000).

Several *SNARE* genes in plants have been shown to be involved in plant resistance against various pathogens (Inada and Ueda, 2014). For instance, HvSNAP34 in barley (*Hordeum vulgare*) was reported to participate in callose deposition during non-host resistance to powdery mildew (Collins *et al.*, 2003). Further research on AtSNAP33, the homologue of HvSNAP34 in *Arabidopsis thaliana*, has revealed the functional SNARE complex PEN1-SNAP33-VAMP721/722 (Wick *et al.*, 2003; Lipka *et al.*, 2008). Recent study shows that SEC11 from *Arabidopsis* modulates PEN1-dependent vesicle traffic by dynamically competing for PEN1 binding with VAMP721 and SNAP33 (Karnik *et al.*, 2013). Tobacco (*Nicotiana tabacum*) *NbSYP132* has been implicated in plant resistance against bacterial pathogen by mediating the secretion of pathogenesis-related protein 1 (Kalde *et al.*, 2007). Another Golgi *SNARE AtMEMB12* was targeted by miR393b* and involved in the accumulation of PR1 (Zhang *et al.*, 2011). By interacting with potyviral 6K2 integral membrane protein, the *Arabidopsis* SNARE protein Syp71 is an essential host factor for successful *Turnip mosaic potyvirus* infection (Wei *et al.*, 2013).

The *Novel Plant SNARE* (*NPSN*) genes are a family of *SNARE* genes that have no homologues in mammalian or yeast genomes (Sanderfoot *et al.*, 2000). Three *NPSN* genes, *NPSN11*, *NPSN12*, and *NPSN13*, were identified in both *Arabidopsis* and rice (*Oryza sativa*) genomes and reported to locate on the plasma membrane (Zheng *et al.*, 2002; Uemura *et al.*, 2004; Bao *et al.*, 2008a). Further research demonstrated that AtNPSN11 in *Arabidopsis* was immuno-fluorescently localized on the cell plate where it interacted with a t-SNARE protein KNOLLE during cytokinesis (Zheng *et al.*, 2002). Interestingly, a recent study illustrated that AtNPSN11 might be involved in one of the two KNOLLE-containing tetrameric SNARE complexes, which jointly mediate membrane fusion during cytokinesis (El Kasmi *et al.*, 2013).

So far, the physiological roles of *NPSN* genes in plant–pathogen interactions have not been well characterised. In this study, three *NPSN*s (*TaNPSN11*, *TaNPSN12*, and *TaNPSN13*) and three plant-defence related *SNARE* homologues (*TaSYP132*, *TaSNAP34*, and *TaMEMB12*) were cloned from common wheat (*Triticum aestivum*) cultivar Xingzi9104 and their possible interactions were investigated by yeast two-hybrid and bimolecular fluorescence complementation (BiFC) assays. The transcriptional regulation of the three *TaNPSNs* and *TaSYP132* in wheat response to *Puccinia striiformis* f. sp. *tritici* (*Pst*) was characterized using qRT-PCR and their functions were further tested by virus-induced gene silencing (VIGS) assay. Localization of TaNPSN11 on vesicle structures near cell membrane toward *Pst* hypha was clarified by immuno-cytochemical methods.

## Materials and methods

### Isolation of cDNA sequences

Several SNARE sequences from NCBI (http://www.ncbi.nlm.nih.gov/) and the IPK barley transcriptome database (http://webblast.ipk-gatersleben.de/barley/), including AtNPSN11 (NP_565800.1), AtNPSN12 (NP_175258.2), AtNPSN13 (NP_566578.1), AtSYP132 (NP_568187.1), AtSNAP33 (NP_200929.1), AtMEMB12 (NP_1998 55.1), NbSYP132 (ABI93942.1), OsNPSN11 (AAU94635.1), OsNPS N12 (AAU94636.1), OsNPSN13 (AAU94637.1), OsSYP132 (BAC79 742.1), OsSNAP34 (NP_001046737.2), OsMEMB12 (NP_00105080 2.1), HvNPSN11 (MLOC_51920.1), HvNPSN13 (MLOC_56623.1), HvSYP132 (AK252235.1), HvSNAP34 (AAP79417.1) and HvMEMB 12 (AK374575), were used to blast the DFCI wheat EST database (http://compbio.dfci.harvard.edu/) and IWGSC wheat genome database (http://www.wheatgenome.org/) to obtain the potential homologues of *NPSN11*, *NPSN12*, *NPSN13*, *SYP132*, *SNAP34*, and *MEMB12* in wheat. Primers for *TaNPSN11*, *TaNPSN12*, *TaNPSN13*, *TaSYP132*, *TaSNAP34*, and *TaMEMB12* were designed (Supplementary Table S1 available at *JXB* online). ORFs of these wheat *SNARE* homologues were amplified from cDNA synthesized using RNA isolated from wheat cultivar Xingzi9104 leaves infected with *Pst* avirulent race (CYR23) 24 hours post-inoculation. The cDNA fragments with 5’ and 3’ UTR for *TaNPSNs* were further cloned using the SMART RACE cDNA amplification kit (Clontech Laboratories Inc., Palo Alto, CA, USA).

### Sequence analysis

The amino acid sequences of six *TaSNARE* homologues cloned in this study were analysed by Pfam (http://pfam.sanger.ac.uk/) for conserved domains or motifs. TargetP 1.1 (http://www.cbs.dtu.dk/services/TargetP/) and TMHMM 2.0 (http://www.cbs.dtu.dk/services/TMHMM/) were used to predict localization and trans-membrane domains. Multiple sequence alignments and the Neighbour Joining tree were created using MUSCLE method by MEGA 5.1 (MEGA, Inc.). The same programme was used to perform 1000 bootstrap cycles to estimate the confidence of the different nodes of the tree.

### Yeast two-hybrid assay

Protein–protein interactions were assayed using the Matchmaker yeast two-hybrid system (Clontech, Mountain View, CA). Fragments of *TaNPSN11*, *TaNPSN12*, *TaNPSN13*, and *TaMEMB12* without the trans-membrane region were inserted into pGBK7 vector as bait constructs. Fragments of *TaNPSN12*, *TaNPSN13*, *TaSNAP33*, *TaSYP132*, and *TaMEMB12* without the trans-membrane region were inserted into pGADT7 vector as prey constructs (primers in Supplementary Table S1 available at *JXB* online). Bait and prey vectors were co-transformed in pairs into the yeast strain AH109. The Leu^+^ and Trp^+^ transformants were isolated and interactions were tested on SD-Trp-Leu-His and SD-Trp-Leu-His-Ade plates as described previously (Cantu *et al.*, 2013; Yang *et al.*, 2013). The control plasmids were provided by the manufacturer.

### Bimolecular fluorescence complementation (BiFC) assay

Bimolecular fluorescence complementation (BiFC) assays were performed in tobacco protoplasts as described by Schütze *et al.* (2009). Based on the positive results from the yeast two-hybrid assays, the full-length TaNPSN11 and TaSYP132 cDNAs were recombined with the N-terminal and C-terminal part of YFP in the vectors pSY736 and pSY735, respectively (primers in Supplementary Table S1 available at *JXB* online). The fusion proteins were co-expressed in tobacco protoplasts using the polyethylene glycol method. Fluorescence was monitored between 24 and 48 hours after transformation using a Zeiss Axiovert 25 fluorescence microscope with the Zeiss YFP filter cube 46HE (excitation, BP 500/25; beam splitter, FT 515; emission, BP 535/30). Co-transformation of TaHSP90-pSY736 and TaRAR1-pSY735 vectors was used as the positive control and recombinant vectors with corresponding empty vectors were co-transformed as negative controls (Cantu *et al.*, 2013). For each treatment, numbers of fluorescent cells and observed cells were counted from ten fields of vision under objective 40×. Calculations for the mean, standard error and ratio of fluorescent cells were performed using SPSS 16.0 software (SPSS Inc.).

### *Pst* inoculation and qRT-PCR assays

Seedlings of wheat cultivar Xingzi9104 were maintained and inoculated with *Pst* avirulent race (CYR23) or virulent race (CYR32) as described by Kang and Li (1984). The wheat plants inoculated with sterile distilled water were used as mock-inoculated controls. The leaves were harvested at 0, 12, 18, 24, 48, 72 and 120 hours post-inoculation (hpi) for RNA isolation. These time points were selected based on previous microscopic studies of the interactions between wheat and stripe rust fungi (Wang *et al.*, 2007; Zhang *et al.*, 2012). All samples were rapidly frozen in liquid nitrogen and stored at –80°C. Three independent biological replications were included for each time point.

The RNAs were isolated using Trizol Reagent (Invitrogen, Carlsbad, CA, USA). First-strand cDNA was synthesized using the GoScript Reverse Transcription System (Promega Corp., Madison, WI, USA). Primers for qRT-PCR were designed (Supplementary Table S1 available at *JXB* online). The wheat elongation factor *TaEF-1a* (GenBank accession number Q03033) was used as an internal reference for qRT-PCR analyses. Primer efficiencies were calculated using five 4-fold cDNA dilutions (1:1, 1:4, 1:16, 1:64, and 1:256) in duplicate as well as checking for amplification in a negative control without cDNA. Dissociation curves ranging from 60 to 94°C were generated for each reaction to ensure specific amplification. The threshold values (Ct) generated from the ABI PRISM 7500 Software Tool (Applied Bio-systems) were used to quantify relative gene expression using the Delta Ct method as described by Chen and Dubcovsky (2012). Transcript levels for all genes and treatments presented in this study are expressed as linearized fold-*EF1a* levels calculated by the formula 2^(*ACTIN* CT – *TARGET* CT)^. The resulting number indicates the ratio between the initial number of molecules of the target gene and the number of molecules of *EF1a* and therefore the Y scales are comparable across genes and treatments. Calculations for the mean, standard error and two-sample *t*-tests for the statistics were performed using SPSS 16.0 software (SPSS Inc.).

### Barley Stripe Mosaic Virus (BSMV)-mediated *TaNPSNs* and *TaSYP132* gene silencing

The virus-induced gene silencing (VIGS) system is an effective reverse genetic tool in barley and wheat (Lee *et al.*, 2012; Wang *et al.*, 2012; Tang *et al.*, 2013). In this study, the plasmids used for virus-induced gene silencing were constructed as described by Holzberg *et al.*, 2002. A cDNA fragment (120bp) of the wheat phytoene desaturase gene *TaPDS* was obtained using RT-PCR. This fragment, in anti-sense orientation, was used to replace the GFP coding sequence in BSMV-GFP (green fluorescent protein) to generate BSMV-TaPDS. Using a similar approach, BSMV-TaNPSN11, BSMV-TaNPSN12, BSMV-TaNPSN13, and BSMV-TaSYP132 were prepared (primers in Supplementary Table S1 available at *JXB* online). Possible RNAi off-target effects of these VIGS constructs were tested by si-Fi software against an established durum wheat transcriptome as previously described (Nowara *et al.*, 2010; Krasileva *et al.*, 2013).

Capped *in vitro* transcripts were prepared from linearized plasmids containing the tripartite BSMV genome using the mMESSAGE mMACHINE® T7 Transcription Kit (Ambion, Austin, TX, USA). Three independent sets of plants were prepared for each of the six BSMV virus constructs (BSMV-00, BSMV-TaPDS, BSMV-TaNPSN11, BSMV-TaNPSN12, BSMV-TaNPSN13, and BSMV-TaSYP132) using a total of 144 seedlings. Another 24 seedlings were Mock inoculated with 1 × Fes buffer as a control. The second leaf of a two-leaf wheat seedling was inoculated with BSMV transcripts by gently rubbing the surface with a gloved finger, maintained in a growth chamber at 23±2°C, and examined for symptoms at regular intervals (Holzberg *et al.*, 2002; Hein *et al.*, 2005; Scofield *et al.*, 2005). Once virus phenotype was observed, the fourth leaf was inoculated with urediospores of Pst avirulent race (CYR23) or virulent race (CYR32). The stripe rust infection types were recorded at 14 days post-inoculation. The fourth leaves were also sampled at 24, 48 and 120 hours post-inoculation for histological observation and RNA isolation. A series of qRT-PCR assays were applied to test the silencing efficiency for each of the BSMV constructs. Using the comparative threshold (2^–ΔΔCT^) method (Livak and Schmittgen, 2001), relative gene expression of *TaNPSN11*, *TaNPSN12*, *TaNSN13*, and *TaSYP132* in wheat plants inoculated with corresponding BSMV-TaNPSN11, BSMV-TaNPSN12, BSMV-TaNPSN13, and BSMV-TaSYP132 was compared with that in BSMV-00, respectively. Calculations for the mean, standard error and two-sample *t*-tests for the statistics were performed using SPSS 16.0 software (SPSS Inc.).

### Histological observations of fungal growth and host response

Wheat leaves infected BSMV were sampled at 24, 48 and 120 hours post-inoculation (hpi) with *Pst*. For further observation, the leaf samples were discoloured using ethanol and acetic acid. For samples collected at 24 hpi, DAB was used to specifically stain the H_2_O_2_ generated at the infection site (Wang *et al.*, 2007). The proportion of H_2_O_2_ accumulation at each infection site was measured using Olympus DP70/DP30BW microscopy with Olympus software (DP-BSW Ver. 02. 03, Olympus). Only infection sites with substomatal vesicle formations were considered as successfully penetrated. For samples collected at 48 and 120 hpi, the proportion of phenolic autofluorogens accumulated at the infection site was measured using Olympus DP70/DP30BW microscopy (excitation filter, 485nm; dichromic mirror, 510nm; barrier filter, 520nm). At least 50 infection sites from each of the five randomly selected leaf segments per treatment were examined.

Rust fungal structures were then specifically stained using Calcofluor White (Sigma Co., USA) as described by Kang *et al.* (1993). The leaf segments were fixed and dehydrated using chloral hydrate for 1 hour. Subsequently, samples were soaked twice in 50% alcohol for 15min followed by three washes with distilled water. The leaf samples were further dehydrated twice with 0.5M NaOH for 10min and washed three times with distilled water. The rust fungal structures were stained using 0.1% Calcofluor White (Tris-HCl buffer, pH 8.5) for 10min, and immediately washed with distilled water for 10min. All samples were preserved in 25% glycerol for observation. The *Pst* hyphae at each infection site were observed using Olympus DP70/DP30BW microscopy (excitation filter, 485nm; dichromic mirror, 510nm; barrier filter, 520nm), and their lengths were calculated by Olympus software (DP-BSW Ver. 02. 03, Olympus). Calculations for the mean, standard error and two-sample *t*-tests for the statistics were performed using SPSS 16.0 software (SPSS Inc.).

### Purification of recombinant TaNPSN11 and western blot

A 564bp *TaNPSN11* cDNA fragment (1–188 aa, 21.19kDa) was amplified and cloned into the pET-28a (+) vector (Novagen, Madison WI, USA). The construct was then transformed into *E. coli* strain BL21 (DE3). The expression of *TaNPSN11* tagged with six histidine residues at the N-terminus was induced using 0.1mM isopropyl β-thiogalactoside at 37°C for 4 hours. For recombinant protein purification, the bacterial cells were pelleted after induction, suspended in coupling buffer (20mM Na_2_HPO_4_ pH 7.4, 0.5mM NaCl, 20mM imidazole) containing 10mg ml^–1^ of lysozyme and subjected to sonication on ice for 30min using a UP100H Vibra Cell sonicator (Hielscher, Teltow, Germany). The resulting lysate was centrifuged at 4°C for 15min at 12 000g and the supernatant was put onto His-Trap HP resin for chromatography using an AKTA Purifier 10 (Amersham Pharmacia Biotech, Uppsala, Sweden). The recombinant protein was eluted with a 20–500mM imidazole gradient in elution buffer (20mM Na_2_HPO_4_ pH 7.4, 0.5mM NaCl, 500mM imidazole). The purified protein was injected into a rabbit to raise anti-TaNPSN11 antibody as described by Harlow and Lane (1999).

For the western blot analysis, protein was extracted from wheat leaves using protein extraction buffer: GTEN (10% (v/v) glycerol, 25mM Tris pH 7.5, 1mM EDTA, 150mM NaCl), 10mM DTT, 2% (w/v) PVPP (polyvinylpolypyrrolidone), and 1 × protease inhibitor cocktail (Moffett, 2011). Wheat leaves were harvested and leaf materials cut off on either side of the middle vein with a razor blade. Next, 1g of leaf tissue was weighed out and placed in a pre-chilled mortar. 2.5ml of extraction buffer was added to each mortar and ground for 1–2min to form a consistent slurry. The slurry was poured into a 2ml Eppendorf tube and spun at full speed in a refrigerated micro-centrifuge for 2min. Supernatant was transferred to a 1.5ml Eppendorf tube and spun for an additional 10min. Supernatant was then separated on a 15% SDS-polyacrylamide gel (SDS-PAGE). The protein was subsequently transferred onto a nitrocellulose membrane using a Semi-Phor Semi-Dry Transfer Unit (Amersham Pharmacia Biotech). The immuno-blot analysis was conducted using the polyclonal antibody raised against the recombinant TaNPSN11 as the primary antibody and a horseradish peroxidase (HRP)-conjugated goat-anti-rabbit IgG (Sigma-Aldrich, St. Louis, MO) as the secondary antibody. The immuno-reactivity was detected using an ECL Western Blotting Substrate kit (Amersham^TM^, UK) and photographed.

### Immuno-cytochemical localization of TaNPSN11

Leaves of seedling wheat were infected with *Pst* avirulent race CYR23 and harvested at 48 hours post-inoculation (hpi). The leaf specimens (approximately 2mm^2^) were excised from the infected tissues, fixed with 3% glutaraldehyde (v/v) in 100mM phosphate buffer (pH 6.8) for 3 hours at 4°C, and washed in the same buffer four times for 15min. The sections were post-fixed with 1% (w/v) O_S_O_4_ in 100mM phosphate buffer (pH 6.8) and washed in the same buffer four times for 15min. Following dehydration in a graded ethanol series, the sections were embedded in LR-white. Ultra-thin sections were cut using a vibratome (TPI, Series 1000) at a 45° angle and mounted on grids.

The grids were incubated three times in 5% (w/v) BSA for 30min and subsequently incubated in a 1:40 000 dilution of the rabbit anti-TaNPSN11 antibody in BSA for 2 hours at room temperature. After washing three times and twice for 10min in 1% BSA and 75mM PBS (pH 7.2–7.4), the grids were incubated for 1h at room temperature in a 1:20 dilution of the colloidal gold (15nm)-conjugated goat-anti-rabbit IgG in PBS. After washing twice for 10min in distilled water, the grids were dried, and the samples were post-stained with uranyl acetate and lead citrate. The sections were examined using a Zeiss-EM10 electron microscope (80kV) (Kang and Buchenauer, 2002; Liu *et al.*, 2010; Wang *et al.*, 2010).

## Results

### Cloning of *TaNPSN*s and plant defence-related *SNARE* homologues

*SNARE* genes from *Arabidopsis*, rice and barley were retrieved and blasted against the DFCI wheat EST database and IWGSC wheat genome database to identify the homologous wheat genes. Six wheat *SNARE* homologues with complete ORFs were cloned from cDNA of wheat cultivar Xingzi9104, and designated as *TaNPSN11*, *TaNPSN12*, *TaNPSN13*, *TaSYP132*, *TaSNAP34*, and *TaMEMB12* (GenBank accession numbers JX104547, JX104548, JX104549, JX104550, JX104551, and JX104552). Subsequent RACE reactions resulted in cloning of cDNA fragments with 5’ and 3’ UTR for the three *TaNPSN*s.

**Fig. 1.**
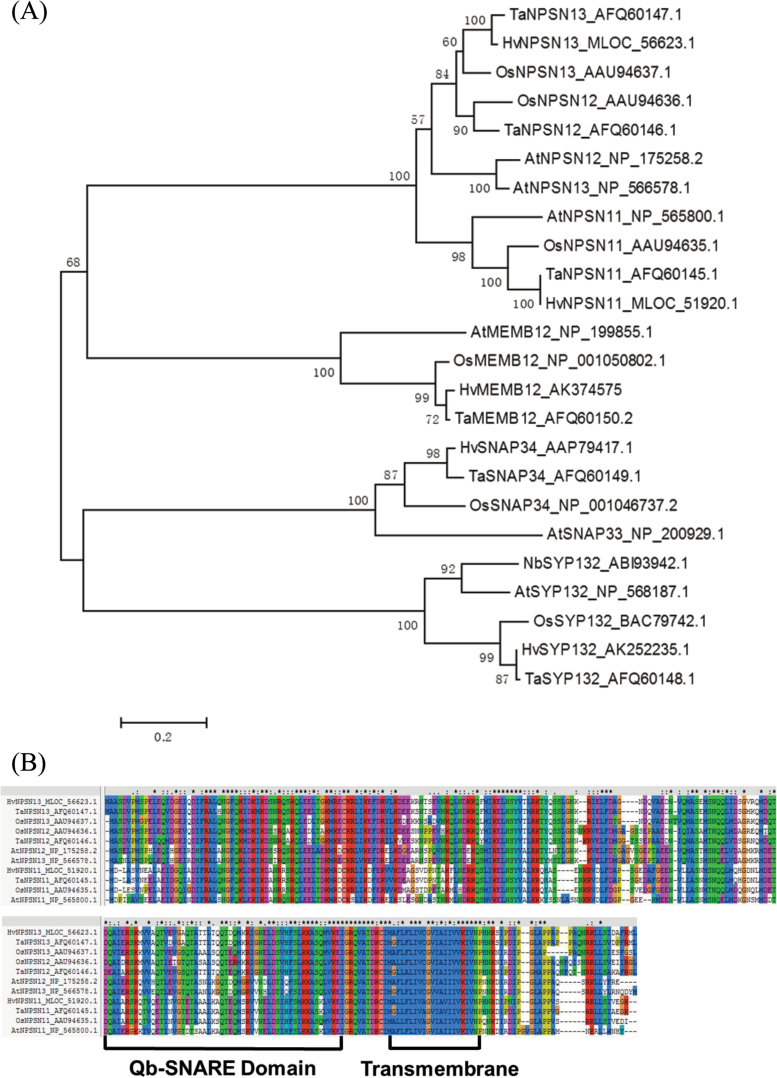
Bio-informatic analysis for all the wheat SNARE homologues cloned in this study. (A) Phylogenetic tree showing all the wheat SNARE homologues cloned in this study and their homologues in other plant species. Multiple sequence alignments and the Neighbour Joining tree were created using the MUSCLE method by MEGA 5.1. Values in the tree nodes indicate bootstrap confidence values based on 1000 iterations. (B) Multi-sequence alignment of TaNPSNs with NPSN proteins from *Arabidopsis*, *Oryza* and *Hordeum*. The amino acid sequences of NPSN proteins shared similar structures, such as the Qb-SNARE domain and trans-membrane region at the C-terminal. Ta, *Triticum aestivum*; Nb, *Nicotiana benthamiana*; At, *Arabidopsis thaliana*; Os, *Oryza sativa*; Hv, *Hordeum vulgare*. (This figure is available in colour at *JXB* online.)

The predicted ORFs of *TaNPSN11*, *TaNPSN12*, *TaNPSN13*, *TaSYP132*, *TaSNAP34*, and *TaMEMB12* encode proteins of 261, 273, 269, 303, 305, and 238 amino acid residues, with molecular weights of 29.0, 30.6, 30.3, 34.2, 33.3, and 26.6kDa, respectively. A multi-sequence alignment and a phylogenetic tree for all the SNAREs in this study were generated (Fig. 1; Supplementary Figure S1 available at *JXB* online). For NPSNs proteins, the conserved Qb-SNARE domain and trans-membrane region at the C-terminus were annotated (Fig. 1B).

### TaNPSN11 interacts with TaSYP132 in yeast two-hybrid and bimolecular fluorescence complementation assays

**Fig. 2.**
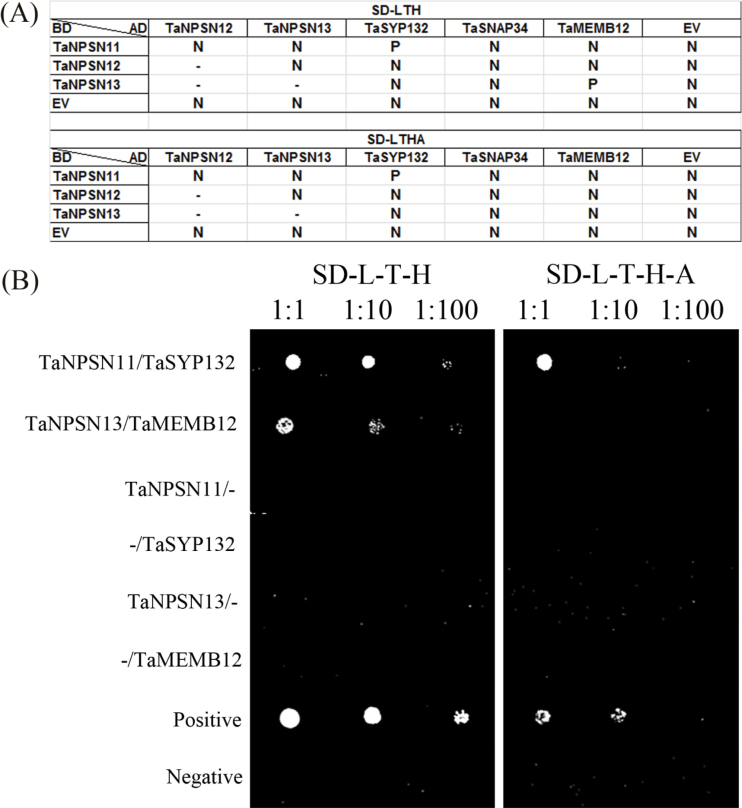
Yeast two-hybrid assay to assess all pairwise interactions between the three TaNPSNs and three plant defence-related SNARE homologues. (A) Yeast transformants co-expressing different bait and prey constructs were assayed on SD-Leu-Trp-His and SD-Leu-Trp-His-Ade plates. (B) TaSYP132 interacted with TaNPSN11 in a yeast two-hybrid assay. The positive and negative controls were provided in the BD Matchmaker Library Construction kit. P, positive; N, negative; EV, empty vector.

Yeast two-hybrid assays were used to characterize all pairwise interactions among the three TaNPSNs and three plant defence-related SNARE homologues in wheat. The results showed that TaSYP132 specifically interacted with TaNPSN11 but not with TaNPSN12 or TaNPSN13. Other combinations showed no interactions (Fig. 2).

**Fig. 3.**
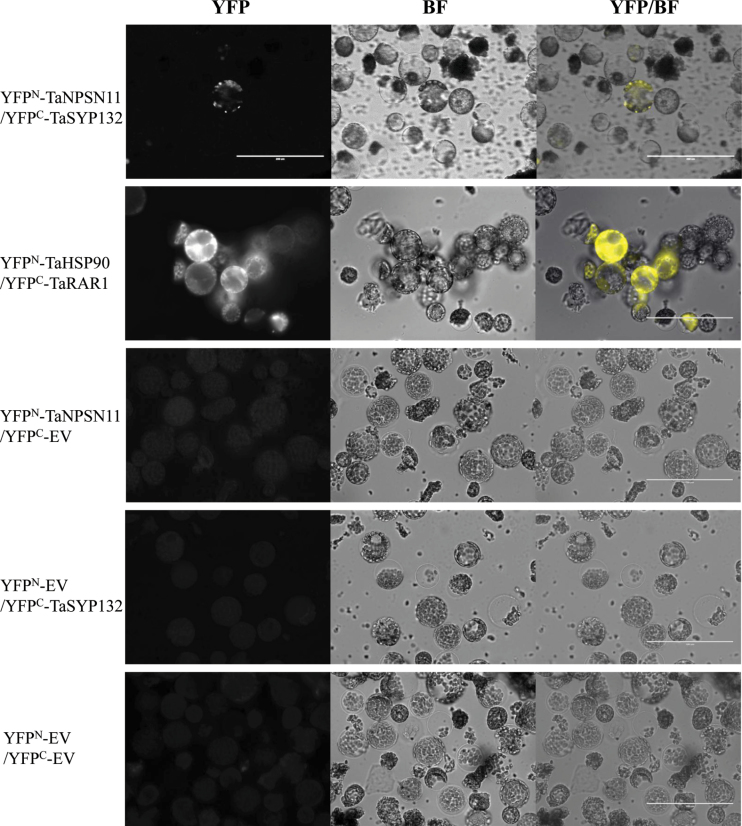
Bimolecular fluorescence complementation (BiFC) assay showing interaction between YFP^N^-TaNPSN11 and YFP^C^-TaSYP132. A BiFC assay was used to visualize protein–protein interaction in tobacco protoplasts. YFP^C^-TaHSP90 and YFP^C^-TaRAR1 were used as positive controls. Negative controls represent each recombinant vector with corresponding non-fused YFP^N^ and YFP^C^ empty vectors. BF, bright field; YFP, yellow fluorescent protein. (This figure is available in colour at *JXB* online.)

To further verify the interaction between TaNPSN11 and TaSYP132 *in planta*, bimolecular fluorescence complementation (BiFC) assays were performed in *Nicotiana benthamiana* protoplasts. YFP fluorescence was reconstituted and punctate localized to the cell membrane when YFP^N^-TaNPSN11 and YFP^C^-TaSYP132 were co-expressed. Stable YFP fluorescence at cytoplasm and nucleus was observed in positive controls using YFP^N^-TaHSP90 and YFP^C^-TaRAR1 (Fig. 3). Although the ratio of fluorescent cells in YFP^N^-TaNPSN11/YFP^C^-TaSYP132 (6%) was lower than YFP^N^-TaHSP90/YFP^C^-TaRAR1 (25%), recombinant vectors with corresponding non-fused YFP^N^ and YFP^C^ empty vectors generated no fluorescence (Table 1).

**Table 1.** Quantitative analysis for bimolecular fluorescence complementation (BiFC) assay

BiFC treatment^a^	Fluorescent cells	Observed cells	Ratio of fluorescent cells
YFP^N^-TaNPSN11/YFP^C^-TaSYP132	1.2±0.1	21.0±1.3	0.06±0.01
YFP^N^-TaHSP90/YFP^C^-TaRAR1	5.7±0.8	21.4±1.7	0.25±0.02
YFP^N^-TaNPSN11/YFP^C^-EV	0	20.0±1.2	0
YFP^N^-EV/YFP^C^-TaSYP132	0	19.8±1.4	0
YFP^N^-EV/YFP^C^-EV	0	20.3±1.2	0

^a^ Co-expression of YFP^N^-NPSN11 and YFP^C^-SYP132 in tobacco protoplast generated punctate fluorescence in plasma membrane. Co-expression of YFP^N^-TaHSP90 and YFP^C^-TaRAR1 was used as a positive control, which had a strong YFP signal in cytoplasm and nucleic. No fluorescence could be observed in negative controls of YFP^N^-TaNPSN11/YFP^C^-EV, YFP^N^-EV/YFP^C^-TaSYP132, and YFP^N^-EV/YFP^C^-EV. For each treatment, numbers of fluorescent cells and observed cells were counted from ten fields of vision under objective 40×. Calculations for the mean, standard error and ratio of fluorescent cell were performed using SPSS 16.0 software. EV, empty vector.

### Transcriptional changes of *TaNPSNs* and *TaSYP132* induced by *Pst* infection

Wheat cultivar Xingzi9104, possibly carrying *YrSK* and *Yr18*, was used as a host plant for *Pst* infection (Liu *et al.*, 2006; Krattinger *et al.*, 2009; Zhang *et al.*, 2012). After inoculating seedling plants of wheat cultivar Xingzi9104 with *Pst* avirulent race CYR23 and virulent race CYR32, the infection type was scored at 14 days post-inoculation (dpi) using the 0–9 scale method (Supplementary Table S2 available at *JXB* online) (Line and Qayoum, 1992). Seedlings of Xingzi9104 exhibit an immune or few necrotic flecks phenotype (Scale 0–1) to *Pst* avirulent race CYR23. Severe sporulation phenotypes (Scale 8–9) could be observed on Xingzi9104 seedlings inoculated with *Pst* virulent race CYR32 at 14 dpi.

**Fig. 4.**
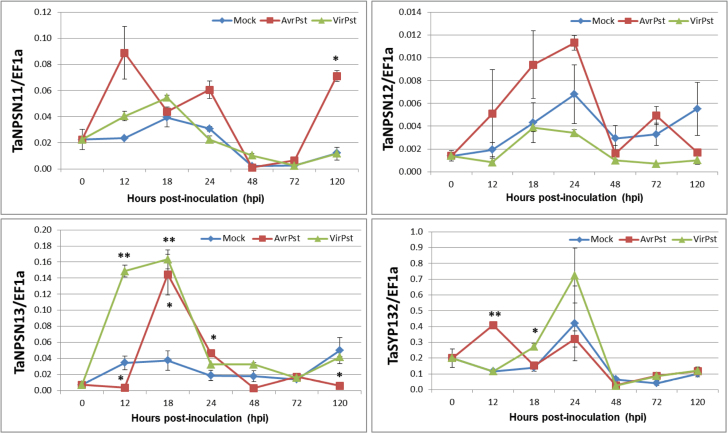
The relative expressions of the three *TaNPSN*s and *TaSYP132* in wheat leaves challenged by *Pst* infection using qRT-PCR assay. Leaf samples were collected at 0, 12, 18, 24, 48, 72 and 120 hours post-inoculation (hpi) with *Pst* avirulent race (CYR23) and virulent race (CYR32), respectively. The Y scale indicates transcript levels relative to endogenous control *EF1a*. The mean, standard error and two-sample *t*-tests were calculated by SPSS 16.0 software with data from three independent biological replicates. (This figure is available in colour at *JXB* online.)

The transcriptional changes of the three *TaNPSN*s and *TaSYP132* induced by *Pst* infections were measured by qRT-PCR (Fig. 4). In leaves inoculated with *Pst* avirulent race CYR23, expression level of *TaNPSN11* showed a significant up-regulation at 120 hours post-inoculation (hpi); *TaNPSN13* was up-regulated at 18 and 24 hpi but suppressed at 12 and 120 hpi; *TaSYP132* was dramatically elevated at 12 hpi. In leaves inoculated with *Pst* virulent race CYR32, *TaNPSN13* was significantly up-regulated at 12 and 18 hpi; *TaSYP132* was strongly induced at 18 hpi. In contrast, *TaNPSN12* did not show any significant inductions by either *Pst* avirulent or virulent races.

### Knocking down *TaNPSN11*, *TaNPSN13*, and *TaSYP132* expression reduced the resistance of wheat to *Pst*

Based on the expression profiles of the three *TaNPSN*s and *TaSYP132* during *Pst* infections, a virus-induced gene silencing (VIGS) system was applied to characterize the role of these *SNARE* homologues during wheat-*Pst* interaction. Four pairs of primers were designed specifically to knock down *TaNPSN11*, *TaNPSN12*, *TaNPSN13,* and *TaSYP132*, respectively. None of the RNAi constructs, except TaNPSN12-VIGS with a weak recognition to Cysteine-rich receptor-like protein kinase 41, was predicted to possess effective off-targets or cross silencing other *SNARE* transcripts in an established durum wheat transcriptome as determined by the si-Fi software (Supplementary Table S3 available at *JXB* online).

**Fig. 5.**
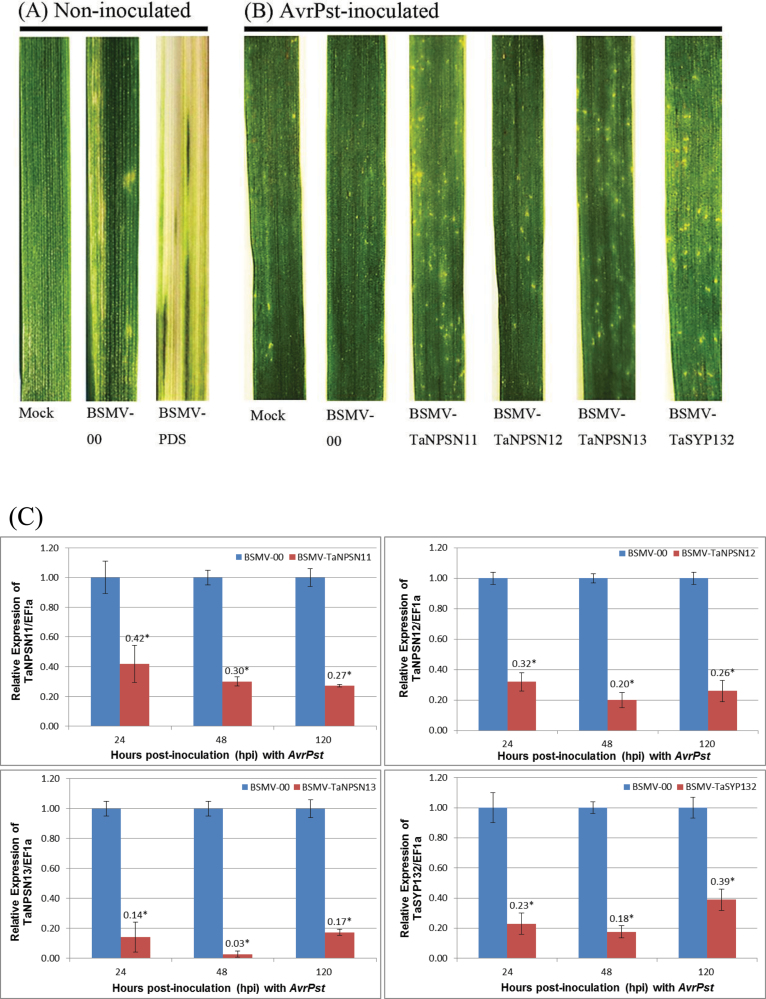
Functional characterization of the three *TaNPSN*s and *TaSYP132* using virus-induced gene silencing (VIGS) assay. (A) mild chlorotic mosaic symptoms were observed on newly expanded third leaves from plants inoculated with BSMV at 9 days post-inoculation (dpi) as a control. Photo-bleaching was evident on the newly expanded fourth leaves in plants pre-infected with BSMV-TaPDS at 14 dpi. (B) After inoculation of the newly expanded fourth leaves with *Pst* avirulent race CYR23. Note the increased number and size of necrotic spots on the wheat leaves pre-infected with BSMV-TaNPSN11, BSMV-TaNPSN13, and BSMV-TaSYP132 compared with those pre-infected with Mock, BSMV-00 and BSMV-TaNPSN12. (C) Relative transcript levels of the three *TaNPSN*s and *TaSYP132* in corresponding knockdown plants assayed by qRT-PCR. Leaf samples were collected from plants pre-infected with BSMV-00, BSMV-TaNPSN11, BSMV-TaNPSN12, BSMV-TaNPSN13, and BSMV-TaSYP132 at 24, 48 and 120 hours post-inoculation (hpi) with *Pst* avirulent race CYR23. The relative expression of *TaNPSN*s and *TaSYP132* was calculated using the comparative threshold (2^–ΔΔCT^) method. The mean, standard deviation and two-sample *t*-tests were calculated by SPSS 16.0 software with data from three independent biological replicates. Mock, wheat leaves treated with 1 × Fes buffer; BSMV, Barley Stripe Mosaic Virus. (This figure is available in colour at *JXB* online.)

All BSMV-inoculated plants displayed mild chlorotic mosaic symptoms at 9 days post-inoculation (dpi), but no obvious defects were observed during further leaf growth. To confirm whether our VIGS system was functioning correctly, BSMV-TaPDS (BSMV vector carrying a segment from the wheat phytoene desaturase gene) was used as the positive control for the gene silencing system, which generates photo-bleaching in the fourth leaves of the inoculated plants (Fig. 5A).

To determine the efficiency of VIGS, qRT-PCR assays were performed on RNA samples extracted from the fourth leaves of wheat seedlings pre-infected with BSMV-00, BSMV-TaNPSN11, BSMV-TaNPSN12, BSMV-TaNPSN13, and BSMV-TaSYP132 at 24, 48 and 120 hours post-inoculation (hpi) with *Pst* avirulent race CYR23. Compared with BSMV-00 control, the abundance of the three *TaNPSN*s and *TaSYP132* transcripts was significantly suppressed to different extents in corresponding knockdown plants (Fig. 5C).

After inoculating seedling plants of wheat cultivar Xingzi9104 with *Pst* avirulent race CYR23, an immune or few necrotic spots phenotype (Scale 0–1) was observed on wheat leaves pre-infected with Mock (buffer inoculated without BSMV), BSMV-00 (empty vector), and BSMV-TaNPSN12, whereas many necrotic spots (Scale 1–2) were observed on leaves pre-infected with BSMV-TaNPSN11, BSMV-TaNPSN13, and BSMV-TaSYP132 at 14 dpi (Fig. 5B; Supplementary Table S2 available at *JXB* online). Thus, the race-specific resistance to the stripe rust fungus was not blocked or eliminated through silencing the expression of the three *TaNPSNs* or *TaSYP132*. Nevertheless, knocking down the expression of *TaNPSN11*, *TaNPSN13* or *TaSYP132* resulted in a reduced resistance of wheat to stripe rust fungus. There were no significant phenotype differences between each treatment when inoculated with *Pst* virulent race CYR32, thus severe sporulation (Scale 8–9) was observed (Supplementary Figure S2 available at *JXB* online).

**Fig. 6.**
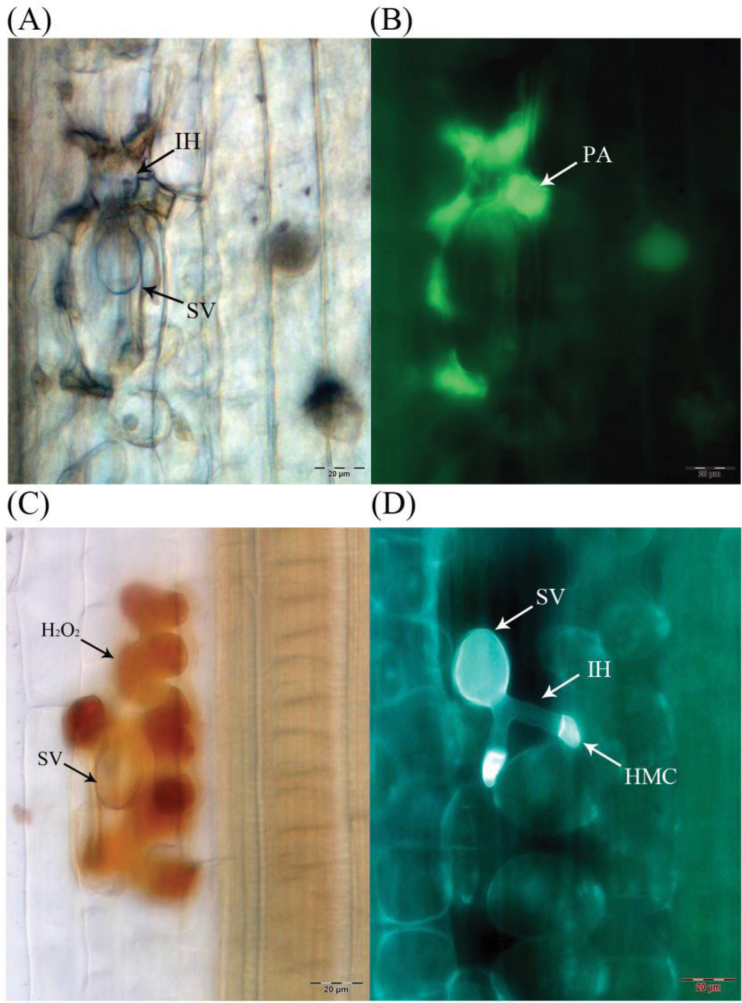
Histological observation of wheat leaves infected with *Pst* avirulent race CYR23 from knockdown plants. (A, B) Infection sites were observed in leaf segments sampled at 48 hours post-inoculation (hpi). Only the infection sites with substomatal vesicle formation were considered as successfully penetrated. The same infection site was observed under both epi-fluorescence and bright field. (C) The accumulation of H_2_O_2_ at the infection site was observed in leaf segments sampled at 24 hpi after DAB staining. (D) *Pst* hypha were observed in leaf segments sampled at 24, 48, and 120 hpi after Calcofluor White staining. BSMV, Barley Stripe Mosaic Virus; SV, substomatal vesicle; IH, initial hyphae; PA, phenolic auto-fluorogens; H_2_O_2_, specific staining of H_2_O_2_ accumulation using DAB; HMC, haustorial mother cell. (This figure is available in colour at *JXB* online.)

To observe the histological changes associated with the enhanced susceptibility to *Pst* in these knockdown plants, leaf segments from at least three plants inoculated with *Pst* avirulent race CYR23 were harvested from each treatment. Accumulation of H_2_O_2_ at the infection site was observed at 24 hpi using microscopy after DAB staining (Fig. 6C). The H_2_O_2_ accumulations in BSMV-TaNPSN13 and BSMV-TaSYP132 pre-infected wheat leaves were significantly (*P* < 0.05) lower than that in BSMV-00 infected leaves (Fig. 6C; Table 2), suggesting the potential involvement of *TaNPSN13* and *TaSYP132* in ROS-mediated plant defence. The phenolic autofluorogen accumulations per infection site in BSMV-TaNPSN11, BSMV-TaNPSN13, and BSMV-TaSYP132 pre-infected wheat leaves were significantly (*P* < 0.01) lower than control at both 48 and 120 hpi (Fig. 6A and B; Table 2), indicating that knocking down *TaNPSN11*, *TaNPSN13*, and *TaSYP132* expression decreases plant defence reactions at infection sites. The leaf samples were further stained using Calcofluor White to enhance the rust hyphal detection (Fig. 6D). The *Pst* hyphal lengths in BSMV-TaNPSN11, BSMV-TaNPSN13, and BSMV-TaSYP132 pre-infected wheat leaves were significantly (*P* < 0.05) longer than those observed in BSMV-00-infected leaves at 24, 48 and 120 hpi (Table 2), indicating that knocking down *TaNPSN11*, *TaNPSN13* and *TaSYP132* expression enhances hyphal penetration of the rust fungus.

**Table 2.** Histological observations during the incompatible interaction between wheat and stripe rust fungus in *TaNPSN11*, *TaNPSN12*, *TaNPSN13* and *TaSYP132*-knockdown plants

	DAB^b^	Phenolic autofluorogens^ c^		Hyphal length^d^		
Treatment^a^	24 hpi	48 hpi	120 hpi	24 hpi	48 hpi	120 hpi
BSMV-00	2.39±1.39a	2.33±0.13a	1.99±0.07a	4.07±0.15a	4.35±0.13a	5.87±0.12a
BSMV-TaNPSN11	2.08±1.04a	1.75±0.06b*	1.44±0.06b*	4.76±0.17b*	5.66±0.12b*	6.26±0.14b*
BSMV-TaNPSN12	2.40±1.28a	2.24±0.08a	1.96±0.07a	4.10±0.18a	4.37±0.11a	5.91±0.13a
BSMV-TaNPSN13	1.99±0.98b	1.91±0.09b*	1.48±0.05b*	4.76±0.17b*	5.73±0.12b*	6.30±0.14b*
BSMV-TaSYP132	1.84±0.89b*	1.71±0.05b*	1.48±0.05b*	4.66±0.16b*	5.52±0.10b*	6.29±0.15b*

^a^ BSMV-00, BSMV-TaNPSN11, BSMV-TaNSN12, BSMV-TaNPSN13, and BSMV-TaSYP132: seedling plants of wheat cultivar Xingzi9104 pre-infected with recombinant BSMV followed by inoculation with *Pst* avirulent race CYR23. hpi, hours post-inoculation.

^b^ Average proportion of H_2_O_2_ accumulation per infection site calculated from at least 50 infection sites (units in 1000 μm^2^ measured by DP-BSW software).

^c^ Average proportion of phenolic autofluorogen accumulation per infection site calculated from at least 50 infection sites (units in 1000 μm^2^ measured by DP-BSW software).

^d^ Average distance from the base of substomatal vesicles to hyphal tips calculated from at least 50 infection sites (units in 10 μm, measured by DP-BSW software).

Calculations for the mean, standard error and two-sample t-tests for the statistics were performed using the SPSS 16.0 software (b*, *P* < 0.01, b, *P* < 0.05).

### TaNPSN11 localizes in vesicle structures near cell membrane towards the infection site of *Pst*

To investigate the distribution of TaNPSN11 during the interaction between wheat and *Pst*, a polyclonal antibody was raised in rabbit against a non-conserved region (amino acids 1–188) of TaNPSN11. A clear band with correct TaNPSN11 size (~21.2 kD) was detected by western blot using protein extracted from *Pst*-infected wheat leaves (Supplementary Figure S3 available at *JXB* online), suggesting that our polyclonal antibody binds to TaNPSN11 (although we cannot rule out the unlikely possibility that it also binds to some other proteins with very similar structure and size).

**Fig. 7.**
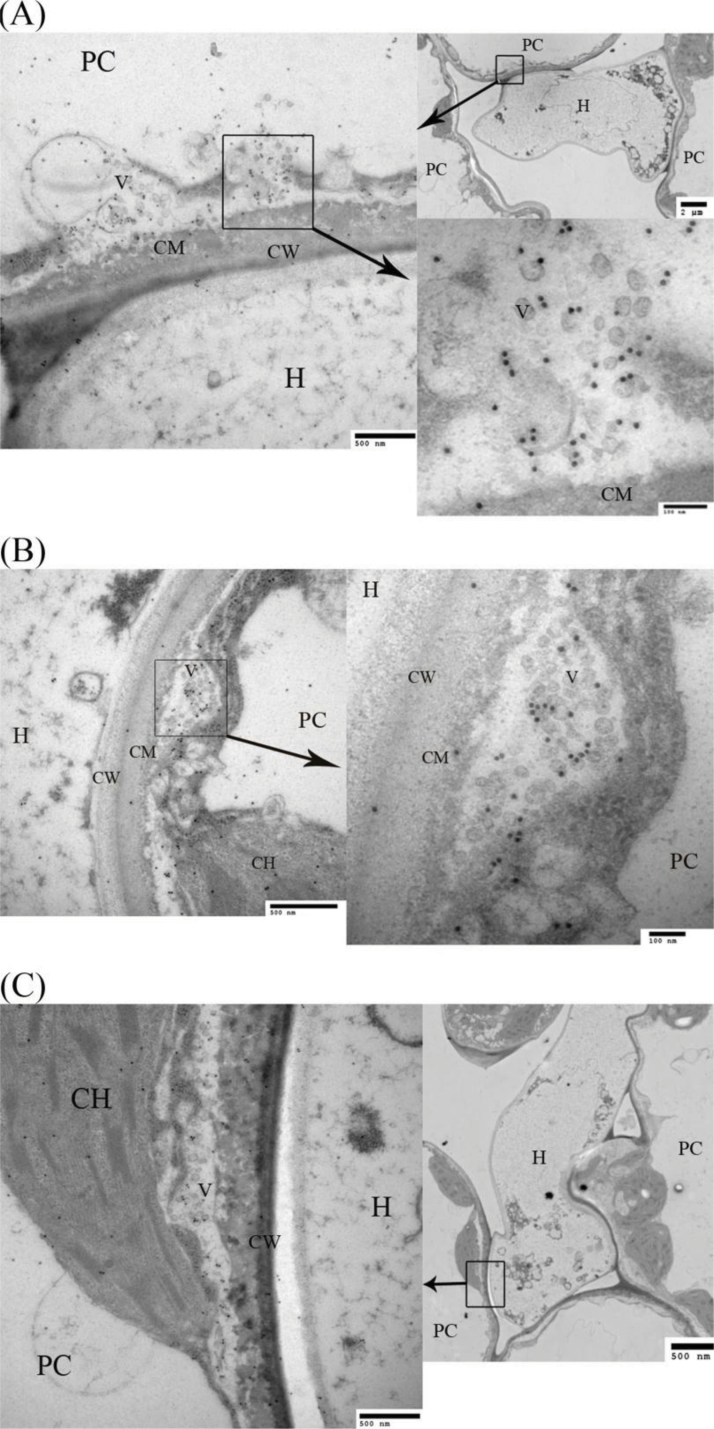
Immuno-cytochemical localization of TaNPSN11 or its structural homologues in wheat leaves inoculated with *Pst* avirulent race CYR23. (A–C) TaNPSN11 was primarily distributed on the vesicle structures near the cell membrane towards *Pst* infection sites. PC, plant cell; H, hyphae; CW, cell wall; CM, cell membrane; V, vesicle; CH, chloroplast.

By immuno-cytochemical localization assays, we observed that TaNPSN11 (or very close structural homologues recognized by the antibody) was primarily (approximately 73%) distributed in vesicle structures towards the intercellular hyphae of *Pst*, especially vesicles near the cell membrane (Fig. 7; Table 3). In addition, the vesicle transport seemed to be activated and redirected to the infection sites (Fig. 7).

**Table 3.** Quantitative analysis for immuno-localisation assay

Distribution^a^	Number of particles	Ratio
Vesicle structure	77.0±7.0	0.73±0.04
Vacuole	6.7±1.3	0.06±0.01
Chloroplast	21.0±6.1	0.05±0.02
Pst hypha	18.1±3.4	0.15±0.02

^a^ The observed immuno-golden particles for NPSN11 antibodies were distributed in vesicle structure, vacuole, chloroplast and Pst hypha. Numbers of particles for each distribution were counted from ten fields of view under magnification of 30 000×. Calculations for the mean, standard error and ratio of each distribution were performed using SPSS 16.0 software.

## Discussion

A total of 54 *SNARE* genes have been identified in *Arabidopsis* including three *NPSN* genes, a number similar to the ones identified in rice (Uemura *et al.*, 2004; Bao *et al.*, 2008b). Using a comparative strategy, we cloned three *NPSN* (*TaNPSN11*, *TaNPSN12*, and *TaNPSN13*) and three plant defence-related *SNARE* homologues (*TaSYP132*, *TaSNAP34*, and *TaMEMB12*) from the wheat cultivar Xingzi9104. The phylogenetic tree shows that the NPSNs, MEMB12, SYP132, and SNAP34 form four well differentiated groups supported by 100% bootstrap values. The NPSN proteins seem to closer to the MEMB12 than to the other groups (68% bootstrap). Within the NPSN cluster, the NPSN forms a well-defined group including both *Arabidopsis* and grass species, suggesting that NPSN11 differentiated before the divergence between the monocots and dicots. The NPSN12 and NPSN13 clusters are well defined in the grasses but separate from the duplication that originated *Arabidopsis* NPSN12 and NPSN13, so these two genes have independent sub-functionalization stories in these two lineages.

Our yeast two-hybrid results show that TaSYP132 interacts with TaNPSN11. Since SNARE complex can be formed with low specificity *in vitro* (Scales *et al.*, 2000), we further validated the interaction in tobacco protoplasts using bimolecular fluorescence complementation (BiFC) assay. The localization of the fluorescent signal confirmed that the interaction punctate occurred at the plasma membrane. Using DUAL membrane protein yeast two-hybrid and immuno-precipitation assays, the interaction between two *Arabidopsis* SNARE proteins Syp71 and Vap27-1 was verified (Wei *et al.*, 2013). The interaction between another *Arabidopsis* SNARE protein SYP121 and SEC11 was characterized in detail by a ratiometric bimolecular fluorescence complementation (rBiFC) assay (Karnik *et al.*, 2013). Since both *TaNPSN11* and *TaSYP132* were expressed with their transmembrane domain in our BiFC assay, the punctate YFP fluorescence at the plasma membrane might indicate that the interaction occurs around the trans-Golgi network (TGN) and plasma membrane (PM). The specificity of TaNPSN11/TaSYP132 interaction also suggests functional differences among NPSN members.In our qRT-PCR assay, expressions of *TaNPSN11*, *TaNPSN13* and *TaSYP132* were differentially induced by either *Pst* avirulent or virulent races, suggesting a possible involvement of these *SNARE* homologues in wheat response to stripe rust infection, which were further characterized by the virus-induced gene silencing (VIGS) assay. Specifically, phenolic auto-fluorescent compounds and H_2_O_2_ accumulations can be used to estimate the strength of the plant defence response toward a pathogen (Holzberg *et al.*, 2002; Wang *et al.*, 2007). The reduced accumulation of phenolic autofluorogens in *TaNPSN11*, *TaNPSN13*, and *TaSYP132*-knockdown plants, together with the significant decrease in hyphal lengths, provides a possible explanation for the more abundant and more visible necrotic areas observed on the surface of *Pst*-infected leaves of the *TaNPSN11*, *TaNPSN13*, and *TaSYP132*-knockdown plants relative to the control. Interestingly, reduced accumulation of reactive oxygen species (ROS) was only observed in the *TaNPSN13-* and *TaSYP132*-knockdown plants, indicating that these two *SNARE* homologues might be involved in the delivery of ROS-related materials. SNARE proteins were normally involved in the plant–microbe interaction by mediating the secretory pathway of functional PRRs or pathogenesis-related (PR) proteins to PM (Beck *et al.*, 2012; Watanabe *et al.*, 2013). Considering the reduced resistance toward *Pst* in *TaNPSN11-* and *TaSYP132*-knockdown plants, as well as their verified protein interaction, we predicted that NPSN11 might complex with SYP132 in the host response to pathogens. In the immuno-localization assays, we observed that TaNPSN11 or its structural homologues was primarily localized on vesicle structures near the plasma membrane towards the *Pst* infection site, and the vesicle transport seemed to be activated and redirected to the infection sites. During cytokinesis in *Arabidopsis*, AtNPSN11 was shown to be localized on the newly formed cell plate using the immuno-fluorescent method (Zheng *et al.*, 2002). Another study illustrated that all three AtNPSN proteins when expressed with GFP-tagging in *Arabidopsis* protoplasts were localized on the plasma membrane (Uemura *et al.*, 2004). Compared with several electron microscopic observation assays on plant vesicles and Golgi apparatus (Coleman *et al.*, 1988; Boevink *et al.*, 1998; McCarthy *et al.*, 2013), we speculate that the bubbly and layered structures observed in our immuno-localization assay are vesicles and Golgi apparatus, respectively. Consequently, our results might provide a snapshot of the position where the TaNPSN11 or its structural homologues mediate vesicle trafficking between the Golgi apparatus and plasma membrane. In conclusion, we predict a dual function for NPSN11 during cytokinesis and plant–pathogen interactions. In *Arabidopsis*, NPSN11 is involved in cytokinesis by forming a KNOLLE-NPSN11-SYP71-VAMP721/722 complex (Zheng *et al.*, 2002; El Kasmi *et al.*, 2013), whereas our results indicate that NPSN11 might also be involved in the transport of plant defence-related materials, possibly through interaction with SYP132. The idea that one SNARE might function in different biological pathways by forming different SNARE complexes is well supported by research concerning *AtSNAP33/HvSNAP34*. Thus, *Arabidopsis* AtSNAP33 interacts with KNOLLE in cytokinesis whereas it (or its barley homologue HvSNAP34) interacted with PEN1 (HvROR2 in barley) in non-host resistance to powdery mildew (Heese *et al.*, 2001; Collins *et al.*, 2003; Lipka *et al.*, 2008). Our study provides initial evidence that these wheat *SNARE* homologues play important roles in vesicle-mediated plant immunity, but the detailed molecular mechanisms and their precise role in wheat defence to stripe rust fungi will require further investigation.

## Supplementary material

Supplementary data can be found at *JXB* online.

Supplementary Table S1. Primers designed and used in this study.

Supplementary Table S2. Scales of stripe rust infection type (IT) in wheat plants.

Supplementary Table S3. RNAi off-target prediction for TaSNAREs-VIGS constructs by si-Fi software.

Supplementary Figure S1. Multi-sequences alignments of TaSNAP33, TaSYP132 and TaMEMB12 with their homologues from other plant species, respectively. Ta, *Triticum aestivum*; Nb, *Nicotiana benthamiana*; At, *Arabidopsis thaliana*; Hv, *Hordeum vulgare*.

Supplementary Figure S2. Phenotypes observed on BSMV pre-infected wheat leaves after inoculation with *Pst* virulent race CYR32. Severe sporulation was observed on wheat leaves pre-infected with Mock, BSMV-00, BSMV-TaNPSN11, BSMV-TaNPSN12, BSMV-TaNPSN13, and BSMV-TaSYP132. Mock, wheat leaves treated with 1 × Fes buffer; BSMV, Barley Stripe Mosaic Virus.

Supplementary Figure S3. Western blot analysis of the polyclonal anti-TaNPSN11 antibody. Anti-TaNPSN11 and HRP-conjugated goat-anti-rabbit IgG antibodies were used as the primary and secondary antibodies in the western blot assay. A clear band with correct TaNPSN11 size (~21.2 kD) was detected by western blot using protein extracted from *Pst*-infected wheat leaves, suggesting our polyclonal antibody could bind to TaNPSN11 or its structural homologues.

Supplementary Data
