# Supplementary material for: Wheat TaNPSN SNARE homologues are involved in vesicle-mediated resistance to stripe rust (Puccinia striiformis f. sp. tritici)
Source: J Exp Bot. 2014 Jun 24;65(17):4807–20. doi: 10.1093/jxb/eru241 (PMC4144766; doi:10.1093/jxb/eru241)
Supplement: Supplementary Data [file supp_65_17_4807__index.html]

Wheat TaNPSN SNARE homologues are involved in vesicle-mediated resistance to stripe rust (Puccinia striiformis f. sp. tritici) — Supplementary Data 

# Wheat *TaNPSN SNARE* homologues are involved in vesicle-mediated resistance to stripe rust (*Puccinia striiformis* f. sp. *tritici*)

## Supplementary Data

Data files

**Files in this Data Supplement:**

- Supplementary Data - Supplementary Data
