# Supplementary material for: Wheat TaNPSN SNARE homologues are involved in vesicle-mediated resistance to stripe rust (Puccinia striiformis f. sp. tritici)
Source: J Exp Bot. 2014 Jun 24;65(17):4807–20. doi: 10.1093/jxb/eru241 (PMC4144766; doi:10.1093/jxb/eru241)
Supplement: Supplementary Data [file supp_eru241_Wang_JXB14_118661_090514_STables_SFigures.pdf]

Supplementary Table S1. Primers designed and used in this study.

	Name	Sequence 5' to 3'	Length
RACE Primers	TaNPSN11-3'RACE-F	GCACAGACAGAGCAAATGAGCAGGGTT	
	TaNPSN11-5'RACE-R	CAACCCTGCTCATTTGCTCTGTCTGTG	
	TaNPSN12-3'RACE-F	ATGCGGTGGTTTCATGCTGCAGTCCT	
	TaNPSN12-5'RACE-R	GAGTGCGCGGAAGATGTCCTGGA	
	TaNPSN13-3'RACE-F	AGCAGCTGGAAGAACTCACTGGGAAGAT	
	TaNPSN13-5'RACE-R	TTCCCAGTGAGTTCTTCCAGCTGCTTG	
qRT-PCR Primers	TaNPSN11-qRT-F	AGAGCAAATGAGCAGGGTT	137 bp
	TaNPSN11-qRT-R	CGACAATGAGAAAAAGCAATC	
	TaNPSN12-qRT-F	TAACACAGCAGACGGAACAAA	147 bp
	TaNPSN12-qRT-R	CTATCAGAGCAAGCAACCCC	
	TaNPSN13-qRT-F	GAAAAGCAATACTTCTGAGGTC	123 bp
	TaNPSN13-qRT-R	ATCCTCTTATTACCAAGGCTAC	
	TaSYP132-qRT-F	AGGAACGGATGGACGATT	148 bp
	TaSYP132-qRT-R	TGCTCACTTCTCCCTGTCT	
VIGS Primers	TaNPSN11-VIGS-F	atattaattaaATCGCCGACATCTTCCGC	369 bp
	TaNPSN11-VIGS-R	tatgcggccgcATGCTGCATTAGCTGCTGGTT	
	TaNPSN12-VIGS-F	atattaattaaCTTGCTCTGATAGTTTTTGGCGT	143bp
	TaNPSN12-VIGS-R	tatgcggccgcGCTTTTGCTGACAACAATCTCCT	
	TaNPSN13-VIGS-F	atattaattaaCGGATTGTTAGTAGCACTTCGGTT	142 bp
	TaNPSN13-VIGS-R	tatgcggccgcTGAATAGACTCGGCTGGTTTTAGG	
	TaSYP132-VIGS-F	atattaattaaGGCAGTATTGGTTGAGGC	161 bp
	TaSYP132-VIGS-R	tatgcggccgcGGAGAAGGATGATGGCGTAG	
Prokaryotic Expression Primers	TaNPSN11-28a-F	gcggatccATGGATTTGGCGTCGGTCA	564 bp
	TaNPSN11-28a-R	gcgtcgacGGAATCCAGCTCATTAACAACCCT	
Bimolecular fluorescence complementation Primers	TaNPSN11-BiFC-F	caccATGGATTTGGCGTCGGTCAAC	786 bp
	TaNPSN11-BiFC-R	CTTGCCCTCTGCAATGGACAAC	
	TaSYP132-BiFC-F	caccATGAACAACCTACTCACCGATTC	906 bp
	TaSYP132-BiFC-R	CTTCTTCCATGGCTGGATAAC	
Yeast Two Hybrid Primers	TaNPSN11-BD-F	gaattcATGGATTTGGCGTCGGTC	619 bp
	TaNPSN11-BD-R	gtcgacCCTGCCTACCAATTTCTTTCAC	
	TaNPSN12-BD-F	gaattcAGCGACGTGCCCATGACC	630 bp
	TaNPSN12-BD-R	gtcgacTACCTGCCGGCCAATCTCTT	
	TaNPSN13-BD-F	ggatccAGCGACGTGCCCATGAGC	621 bp
	TaNPSN13-BD-R	ctgcagTGCAACCTGACGACCAATCTCT	
	TaMEMB12-BD-F	gaattcCTGTGGCGCTCCATCCCC	405 bp
	TaMEMB12-BD-R	ggatccGACCCGGTGCCGCTTCTC	
	TaNPSN12-AD-F	gaattcAGCGACGTGCCCATGACC	630 bp
	TaNPSN12-AD-R	ggatccTACCTGCCGGCCAATCTCTT	
	TaNPSN13-AD-F	atcgatAGCGACGTGCCCATGAGC	621 bp
	TaNPSN13-AD-R	ggatccTGCAACCTGACGACCAATCTCT	
	TaMEMB12-AD-F	gaattcCTGTGGCGCTCCATCCCC	405 bp

	TaMEMB12-AD-R	ggatccGACCCGGTGCCGCTTCTC	
	TaSNAP33-AD-F	gaattcATGAGCGCCACGAGGTCG	741 bp
	TaSNAP33-AD-R	atcgatATCCTGCTTTGCCTTCTCCACC	
	TaSYP132-AD-F	gaattcATGAACAACCTACTCACCGATT	825 bp
	TaSYP132-AD-R	ggatccTCTCGAGTTTTTCTGCAGCT	

Supplementary Table S2. Scales of stripe rust infection type (IT) in wheat plants

Scale	*Description*
0	No Visible infection
1	Necrotic/chlorotic flecks without sporulation
2	Necrotic/chlorotic stripes without sporulation
3	Necrotic/chlorotic stripes with trace sporulation
4	Necrotic/chlorotic stripes with light sporulation
5	Necrotic/chlorotic stripes with intermediate sporulation
6	Chlorotic stripes with moderate sporulation
7	Chlorotic stripes with abundant sporulation
8	Stripes without chlorosis, moderate sporulation
9	Stripes without chlorosis and abundant sporulation

Supplementary Table S3. RNAi Off-target prediction for TaSNAREs-VIGS constructs by si-Fi software.

RNAi constructs[a]	Database ID	All Hits	Effective Hits	Annotation
**TaNPSN11-VIGS**	Td-k56_contig_29261	129	69	AFQ60145.1_NPSN11 [Triticum aestivum]
	Td-k26_contig_12140	109	49	AFQ60145.1_NPSN11 [Triticum aestivum]
	Td-k61_contig_12194	108	50	AFQ60145.1_NPSN11 [Triticum aestivum]
	Td-k64_contig_39630	0	1	BAN15014.1_SAR DNA binding protein [Bromus inermis]
	Td-k56_contig_36112	0	1	BAN15014.1_SAR DNA binding protein [Bromus inermis]
	Td-k46_contig_32644	0	1	BAN15014.1_SAR DNA binding protein [Bromus inermis]
	Td-k61_contig_32539	0	1	BAN15014.1_SAR DNA binding protein [Bromus inermis]
**TaNPSN12-VIGS**	Td-k26_contig_20993	61	29	AFQ60146.1_NPSN12 [Triticum aestivum]
	Td-k51_contig_20118	61	29	AFQ60146.1_NPSN12 [Triticum aestivum]
	Td-k61_contig_61071	25	12	EMT22028.1_Cysteine-rich receptor-like protein kinase 41 [Aegilops tauschii]
**TaNPSN13-VIGS**	Td-k64_contig_28463	35	33	EMS52760.1_Plant SNARE 13 [Triticum urartu]
**TaSYP132-VIGS**	Td-k56_contig_7091	59	61	ABG75752.1_putative syntaxin-related protein [Triticum aestivum]
	Td-k51_contig_57547	40	29	ABG75752.1_putative syntaxin-related protein [Triticum aestivum]
	Td-k56_contig_73591	22	26	ABG75752.1_putative syntaxin-related protein [Triticum aestivum]
	Td-k51_contig_52092	3	5	ABG75752.1_putative syntaxin-related protein [Triticum aestivum]
	Td-k46_contig_8512	2	6	BAJ90403.1_predicted protein [Hordeum vulgare subsp. vulgare]

[a] None of the RNAi constructs, except TaNPSN12-VIGS with a weak recognition to Cysteine-rich receptor-like protein kinase 41, was predicted to possess off-targets or cross silencing of other SNARE transcripts in an established durum wheat transcriptome as determined by the si-Fi software.

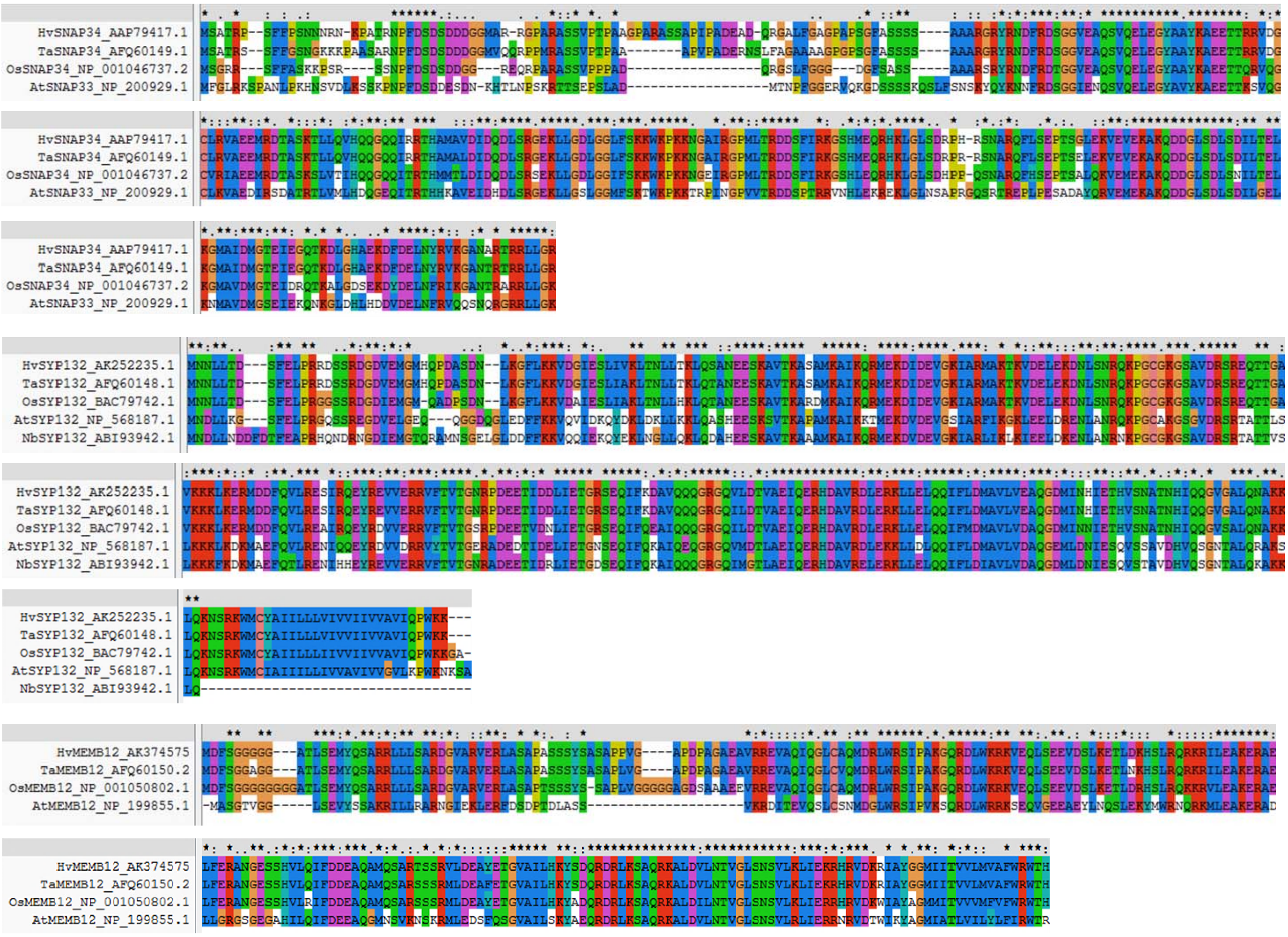

Supplementary Figure S1

VirPst-inoculated

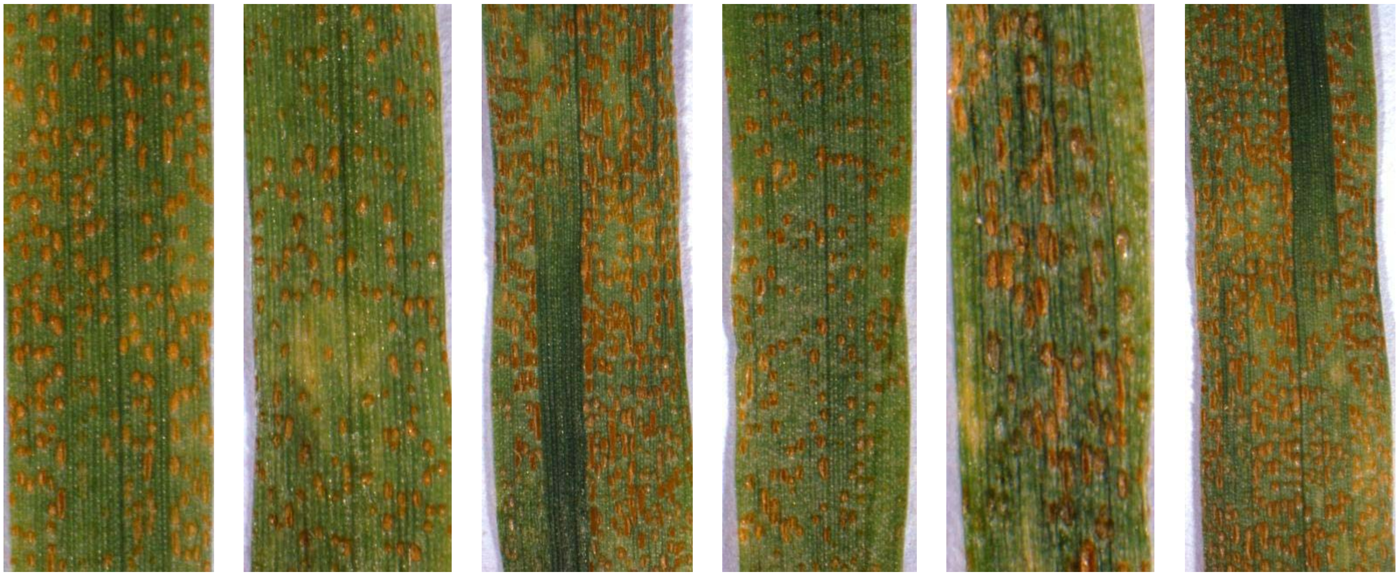

Mock BSMV-00 BSMV-TaNPSN11 BSMV-TaNPSN12 BSMV-TaNPSN13 BSMV-TaSYP132

Supplementary Figure S2

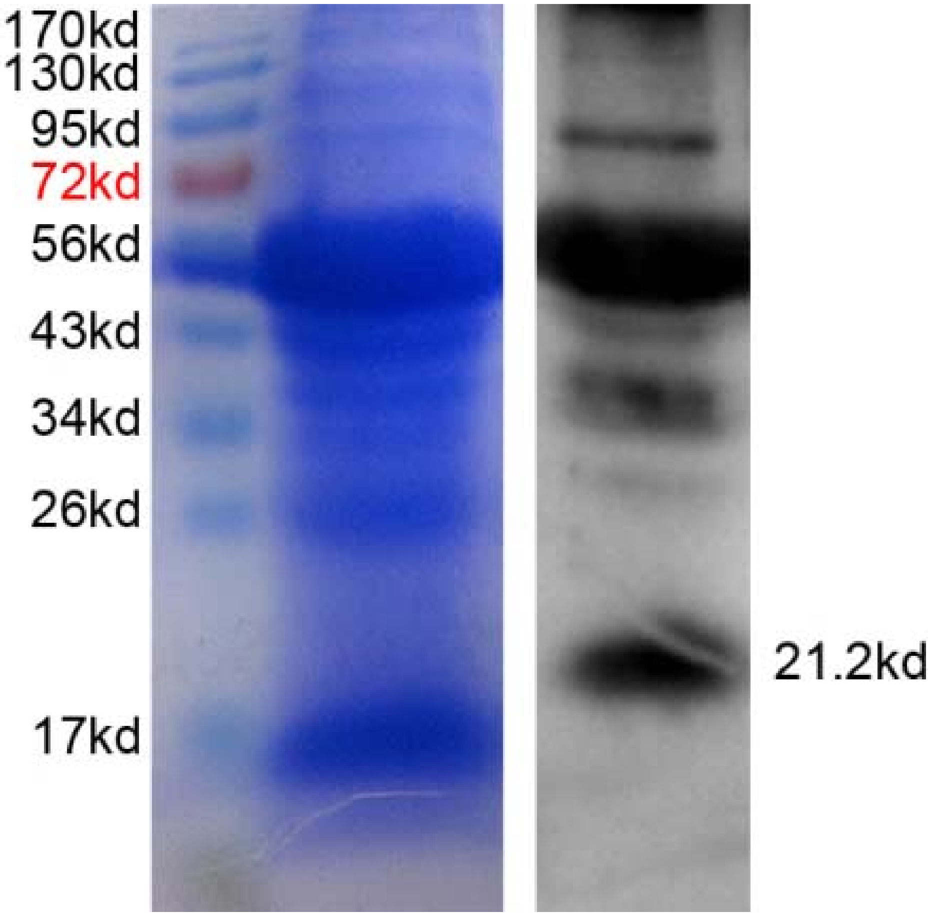

Supplementary Figure S3
